# Dr. Arthur T. O’Hara

**Published:** 1905-02

**Authors:** 


### OBITUARY.

Dr. Arthur T. O'Hara, of Buffalo, was found dead in his quarters at the quarantine hospital, East Ferry street, Sunday morning, January 1, 1905. His sudden death was attributed to cardiac rheumatism, a disease which had afflicted him for some time. Dr. O'Hara was 43 years old and for thirteen years he had been superintendent of the Buffalo quarantine hospital. This practically isolated him, thus depriving him of the ordinary social and professional privileges accorded physicians in general. He, however, became a specialist in contagious diseases, his opinion often being sought in doubtful or obscure manifestations. He was held in high esteem by the health authorities, not only because of the faithful discharge of his exacting duties but, further, because of his skill in his chosen field and of his professional uprightness. His body was incinerated at the Buffalo crematory.

